# Ethnoecological knowledge of ticks and treatment of tick-borne diseases among Maasai people in Northern Tanzania

**DOI:** 10.14202/vetworld.2015.755-762

**Published:** 2015-06-20

**Authors:** John Kioko, Julia Baker, Avery Shannon, Christian Kiffner

**Affiliations:** 1 Center For Wildlife Management Studies, School for Field Studies, P.O. Box 304, Karatu, Tanzania; 2 Biochemistry Program of Biochemistry, Vassar College, Poughkeepsie, New York, USA; 3 Department of Biology, University of Richmond, Virginia, USA

**Keywords:** ethnoveterinary medicine, Maasai, Tanzania, tick-borne diseases, tick ecology

## Abstract

**Aim:**

The aim of this study was to understand traditional knowledge of tick ecology and remedies for tick-borne diseases (TBDs) among the Maasai people in northern Tanzania.

**Materials and Methods:**

Semi-structured interviews were conducted among specific groups likely to be knowledgeable about tick ecology and TBDs in livestock among the Maasai people.

**Results:**

A total of 25 plant species belonging to 18 families were used to treat 8 different TBDs of livestock. Most of the plant species used were of *Fabaceae* and *Burseraceae* families. *Aloe volkensii*, *Cissus grandifolia*, and *Terminalia brownii* were the most commonly used plant species. The major plant growth form used was trees, while stems and bark were the main plant parts used. Most treatments were taken orally.

**Conclusion:**

Maasai people have substantial knowledge on tick ecology exemplified by their ability to differentiate between different tick species and the range of remedies for each of the TBDs. Because traditional ethnoveterinary remedies are frequently utilized, their effectiveness should be further investigated.

## Introduction

The Maasai traditionally live a pastoral lifestyle in parts of East Africa [1] and are known to have a strong culture with customs and norms manifested in their knowledge of traditional system of livestock keeping. In East Africa, tick infection and tick-borne diseases (TBDs) are prevalent and a severe constraint to livestock keepers [2]. In some pastoral areas of Tanzania, calf mortality of cattle reaches 40-80% due to East Coast Fever (ECF), amounting to yearly losses of about USD 43 million [3]. In the Ngorongoro area in northern Tanzania, TBD infection rate is 85.6% [4]. While Maasai people experience continuous and seasonal outbreaks of TBDs [5], their knowledge on tick ecology and epidemiology is not well documented [4].

Traditional knowledge is critical in disease prevention, control, and treatment, and may enhance cost-effective management of TBDs in livestock [6]. Past studies suggest that the treatment seeking behavior in rural areas is closely related to their cultural knowledge, with people often seeking the locally available options first [7]. Furthermore, there is continuing loss of traditional knowledge among the Maasai people as they adopt new lifestyles [5]. This is unfortunate because traditional knowledge on TBDs may help enrich modern pharmacology [8]. The objective of this study was to examine and document the ethno-ecological knowledge of ticks and TBDs among the Maasai people of Northern Tanzania. The specific objectives were to common tick species and associated TBDs in livestock, identify plants and plant parts used to prepare remedies for TBDs, and document how the remedies are prepared and administered among the Maasai of Northern Tanzania.

## Materials and Methods

### Ethical approval

In order to protect the rights and welfare of the interviewees, guidelines based on the School For Studies Institutional Review Board (IRB) - IRB-TZ-01-13 were followed.

### Study area

The study was undertaken in Monduli district within the villages of Esilalei, Losirwa, and Eselela, within Arusha Region in Northern Tanzania. The area is a semi-arid savannah, with an average annual rainfall of 650 mm [9]. We worked in pastoral areas bordering Lake Manyara National Park and Manyara Ranch, that are commonly used by dispersing and migrating wildlife [10]. The ecosystem is rich in wildlife diversity with about 350 birds, 290 reptiles, and 40 amphibian species [11] and a host of large mammal species. The area is mainly used by the Maasai people for livestock grazing [11-13].

### Sampling procedure

Interviewees were selected based on a purposive sampling procedure [14]. Twenty three livestock keepers likely to be knowledgeable in tick ecology, and TBDs were identified by asking elders in each village. These included 15 men, 5 women, and 3 individuals known to specialize in traditional veterinary medicine. A livestock veterinarian of the Maasai tribe was consulted so as to corroborate some of the information, e.g., the English names for specific TBDs and names of tick species. Among these groups, semi-structured [15] and informal interviews [16], were undertaken within a period of 10 days.

Authors 2 and 3 conducted the interviews assisted by a translator well versed in English and Maasai (*maa*) language. The interviewees were shown pictures of ticks [17] and requested to give the *maa* tick species name and associated disease known to be transmitted by that tick species. Other information collected during the interview included: the preferred habitat of each tick species, tick species-specific attachment sites, seasonal occurrence, and the main host species affected. In addition, we asked about details on possible treatment methods. We asked what kind of plant and other parts/products were used, how the medication was prepared and applied, and how available and effective the treatments were. Plant walks in the villages were undertaken for 2 days to collect and identify the plants used for TBD treatment. A Likert scale rating [18], where (1 = common 2 = relatively common [required searching in the village] 3 = rare [rare in the area and has to be sought far]) was used to characterize the availability of each plant. The perceived tick remedy effectiveness was classified as: 1 = rarely effective, 2 = moderately effective, 3 = very effective.

### Statistical analysis

A checklist of plant species used for TBD remedies was generated and taxonomic classification undertaken [19]. A Chi-square goodness of fit test was used to determine differences in perceived level of effectiveness and species availability for treatment of TBDs.

## Results

### Traditional knowledge on tick ecology among the Maasai

A total of 4 common *maa* names were used to describe six species of ticks found in the area. Ticks were said to prefer shaded areas, grass tops, and bark crevices and were said to be most abundant in the dry season. The local tick control methods mentioned included pasture burning (5%), hand picking (15%), topical application of kerosene (70%), and cow dung to remove ticks (10%). However, pasture burning (5%) was said to be practiced in limited cases as it reduces the available grazing land (Table-1). All interviewees relied on the use of acaricides for tick control.

**Table-1:** Ecological knowledge of ticks among the Maasai in Northern Tanzania.

Tick species	English tick name	Local tick name	Preferred habitat	Observed attachment site	Main season	Animal affected
*Amblyomma variegatum; Hyalomma truncatum*	Bont tick; bont-legged tick	Armaheripus	Under trees, under grass	Anus, groin, dewlap, udders	Dry season	Cattle, goat, sheep
*Rhipicephalus decoloratus*	Blue tick	Endenuri/Armaherikima	Top of tall grass, top of short grass	Anus, ears, eyes (softer skin areas)	Dry season	Cattle, goat
*Rhipicephalus appendiculatus;* *Rhipicephalus* spp.; *Rhipicephalus evertsi evertsi*	Brown tick; red tick; red-legged tick	Armaheriodo	Top of tall grass, bark of trees	Anus, base of tail, dewlap	Dry season	Cattle, goat, sheep

Eight different diseases associated with ticks were noted. Their *maa* names were given as Anaplasmosis (*Oltikana oirobi*), ECF (*Oltikana oirowa*), sweating sickness (*Oltikana Looholo*), heartwater (*Ormilo*), corridor disease (*Engeeya Osero*), red water (*Olodokulak*), lumpy skin disease (LSD) (*Ormoko*), and Brucellosis (*Orkibiroto*). When asked about the vector for each of the TBDs, Anaplasmosis (62%), ECF (56%), Sweating Sickness (50%), and heartwater (22%) were highly associated with ticks. Corridor disease (18%), Babesiosis (red water) (9%), LSD (5%), and Brucellosis (5%) were rarely associated with ticks (Table-2).

**Table-2:** Perceived causes and tick associations with TBDs among Maasai in Northern.

Disease	Tick vector species (17)	Disease association (%) with ticks by respondents	Disease association (%) with the known vector tick species by respondents	Other reported causes of TBD
Anaplasmosis	Blue tick	62	86	New grass, grass around rivers
Brucellosis	Blue tick, brown ear tick, bont tick	5	100	Other diseases (anaplasmosis, ECF, bovine pneumonia), starvation
Corridor disease	Brown ear tick	18	0	Wild animals, tsetse flies, grasses of deep forest
ECF	Brown ear tick	56	40	Green pastures, new grass
Heartwater	Bont tick	22	40	Insect in brain, wild animals, sunlight
Lumpy skin disease	Blue tick, brown ear tick, bont tick	5	100	Red-billed oxpecker, wild animals, the bush
Red water	Blue tick	9	0	Dirty water, tsetse flies, olopito grass, other livestock
Sweating sickness	Bont-legged tick	50	33	

ECF=East Coast Fever, TBD=Tick borne diseases

When asked about the specific tick species that transmitted the pathogens causing each TBD disease, Brucellosis (100%), LSD (100%), and anaplasmosis (86%) were highly associated with the known tick vector (Table-2). None of the respondents knew the tick species that transmitted the pathogens causing corridor and red water diseases. Other agents believed to cause TBDs included new grass, insects, birds such as oxpeckers, dirty water, and other diseases.

### Important medicinal plants for treatment of TBD

The interviewees reported 25 plant species, from 18 families and 21 genera to be used to treat the 8 different TBDs. The most common plant families used were *Fabaceae* (25%) and *Burseraceae* (8%). Most of the treatments (60%) were from trees, compared to shrubs (28%) and herbs (12%). Stems (33%) and bark (29%) were the most used plant parts when compared to leaves (16%) and roots (14%), fruits (14%), sap (2%), and branches (2%). Most of the plants were said to be readily available locally or in the nearby foothills, however, some of the herbs were only available during the rainy season. The most important plant reported to be used was *Aloe volkensii*, and was used to treat 6 of the 8 TBDs (Table-3). The next most important plants were *Cissus grandifolia* and *Terminalia brownii*, which were used to treat 4 of the 8 TBDs investigated.

**Table-3:** TBD and associated plant species used for treatment by Maasai in Maasai in Northern Tanzania.

Disease	Plant scientific name	Plant *maa* name	Parts used, preparation, dosage, and effectiveness
Anaplasmosis	*Cissus grandifolia*	*Endijai*	Feed the animal a handful of pounded plant, 1 time (×)/day, for 2-3 days
	*Cissus grandifolia*	*Endijai*	Feed the animal a handful of pounded plant for 1 time (×)/day
	*Cissus grandifolia*	*Endijai*	Chop stalk and boil, cool, and feed to animal, 3 L, 1×/day for 3 days
	*Cissus grandifolia*	*Endijai*	Mix plant stalk pound and soda ash, feed small handful to animal 1×/day for 5 days, rarely effective
	*Argemone mexicana*	*Olemoloko*	Pound stem, mix in soda ash with a little of water, feed to cow 0.25 kg/1×day for 3 days, very effective
	*Aloe vokensii*	*Osukuroi*	Boil leaves and cool, give solution to animal, 1-4 L 1×/day for 2-5 days, moderately effective
	*Aloe vokensii*	*Osukuroi*	Chop leaves and boil, add soda ash, cool, and feed solution to animal, every effective
	*Commiphora spp.*	*oldemwai*	Squeeze out sap from stem and apply topically on the ticks, very effective at removing tick
Brucellosis	*Ficus sycomorous*	*Orng’aboli*	Boil bark and let the mix stay for 2 days, then feed the solution to the animal 1 L/day
	*Adansonia digitata*	*Olmasera*	Boil bark and give solution to cattle, 350 mL, once, very effective
	*Acacia drepanolobium*	*Eluai*	Boil or soak bark, feed solution to animal, 1 L a day for 2-4 days, very effective
	*Adansonia digitata and Acacia nubica*	*Olmasera and Oldepe*	Boil together bark of both trees, give solution to animal 1 L 1×day for 3 days, very effective
Corridor disease	*Cordia sinensis*	*Edorko*	Chop and boil roots, give solution to animal, 2×daily for 3 days, very effective
	*Terminalia brownii*	*Orbukoi*	Boil/soak bark, give solution to animal, rarely effective
	*Lannea schweinfurthii*	*Orpadwa*	Smash roots and collect the juice, feed to animal, 1×day for 3 days, moderately effective
	*Aloe vokensii*	*Osukuroi*	Boil leaves, give solution to animal 1×day for 2-3 days, volume based on body size, moderately effective
	*Aloe vokensii*	*Osukuroi*	Chop leaves and boil, add soda ash, cool, feed solution to animal, 1 L 1×day for several days, rarely effective
ECF	*Dicrostachys cinerea*	*Endendundulu*	Boil roots and add soda ash, inject solution up nose 2×day for 4 days, very effective
	*Aloe vokensii*	*Osukuroi*	Chop leaves and boil, add soda ash, cool, feed solution to animal, 1×day for several days, rarely effective
	*Aloe vokensii*	*Osukuroi*	Boil leaves, give animal 2 L of solution 1×day until recovered, moderately effective
	*Cissus grandifolia*	*Endijai*	Boil and give solution to animal, take hot iron and burn swollen glands, 1-2 L 1×day until recovered, rarely effective
	*Acacia mellifera; Dalbergia melanoxylon*	*Oiti; Oltiasika*	Boil bark from tree and feed solution to animal, 2 L 2×day for 4 days, moderately effective
	*Kigelia africana*	*Aldarapoi*	Take sausage fruit, cut up, boil, give solution to animal, 1.5 L 1×day for 4 days, very effective
	*Aloe vokensii; Cissus grandifolia*	*Osukuroi; Endijai*	Boil leaves and stalk together and let cool, feed solution to animal 1 L 2×day for 3-4 days, moderately effective
	*Terminalia brownii*	*Orbukoi*	Orbukoi bark boiled/soaked in water and fed to animals, 350 mL, 2×day, until calf recovers, moderately effective
	*Cissus grandifolia*	*Endijai*	Mix alkaline with plant stalk and feed animal a small handful of the soda ash/plant mash, rarely effective
Heart water	*Cissus grandifolia*	*Endijai*	Chop up and boil, give 1 L of solution 1×day, every other day, for 3 days, rarely effective
	*Albizia gummifera; Cissus grandifolia*	*Ormokutan; Endijai*	Take leaves and roots of tree, stalk of endejai, boil together into solution, 1.5 L on day 1 and 1.5 L on day 3, rarely effective
	*Terminalia brownii*	Orbukoi	Chop up and boil, give 1 L of solution 1×day, every other day, for 3 days, rarely effective
	*Sisala agave*	Olkatani	Chop up and boil leaf, give 1 L of solution every other day, for 3 days, rarely effective
	*Solanum incanum*	Endulelei	Take roots and ripe fruits of sodom apple, boil and mix with tobacco powder, inject solution up nose, rarely effective
	*Commiphora zimmermanii*	Arpande	Boil bark and give solution to animal, 350 mL 1×day for 4 days, moderately effective
	*Cissus grandifolia*	Endijai	Boil stalk and feed to animal 350 mL of solution 1×daily until recovered, very effective
	*Musa acuminata*	Ormangulai	Feed solution to animal until condition improves, rarely effective
	*Terminalia brownii; Aloe vokensii*	Orbukoi; Osukuroi	Boil or soak leaves overnight and give solution to animal, 1 L 2×day for 5 days, very effective
	*Terminalia brownii*	Orbukoi	Boil bark and let cool, give solution to animal, 1-2 L, 1-2×day, for 3-10 days, very effective
	*Salvadora persica*	Oremit	Scrape roots and grate into warm water, stir, and feed foam solution produced to animal once, moderately effective
	*Terminalia brownii*	Orbukoi	Boil bark and wait for solution to cool; add soda ash, feed animal 700 mL of solution 1×day for 2 days without water, moderately effective
Sweating sickness	*Aloe vokensii*	Engaramalasey	Grind up roots and put in warm water; swirl until foamy and bubbles form, bubble applied to tick areas once, very effective
	*Azadirachta indica*	Engusero	Take leaves and bark, give juice from them to animal, 2×day for 4 days, very effective
	*Sisala agave*	Olkatani	Chop up and soak in water, give solution to animal, 0.5 L 1×day for 3 days, moderately effective
	*Aloe vokensii*	Osukuroi	Boil leaves into solution, give animal 0.5 L 1×day for 2-3 days, moderately effective
LSD	*Commiphora sp.*	Oldemwai	Apply juice from stems directly to backs 1×for 5-7 days (juice can be bought), very effective
	*Aloe vokensii*	Osukuroi	Take fresh leaves, grind them up, mix with water, put ointment on infection, once a day for 4 days, very effective
	*Ricinus communis*	Ordulai	Pound leaves and apply paste to wound site, once, very effective
	*Opuntia vulgaris*	Orpopongi	Burn and crush up coals and press paste onto wounds, apply 1×daily for 7 days, moderately effective
	*Albizia gummifera*	Ormokutan	Burn and crush up coals and press paste onto wounds, apply 1×daily for 7 days, moderately effective
Red water	*Commiphora zimmermanii*	Arpande	Boil bark and give solution to animal, 350 mL 1×day for 4 days
	*Cissus grandifolia*	Endijai	Boil stalk and feed to animal 350 mL 1×daily until recovered, very effective
	*Musa* spp.	Olmaringu	Feed to animal until condition improves
	*Terminalia brownii; Aloe vokensii*	Orbukoi; Osukuroi	Boil or soak leaves overnight and give solution to animal, 1 L 2×day for 5 days
	*Terminalia brownii*	Orbukoi	Boil bark and let cool, give to animal, 1-2 L, 1-2×day, for 3-10 days
	*Salvadora persica*	Oremit	Scrape roots and grate into warm water, stir and feed foam produce to animal once
	*Terminalia brownii*	Orbukoi	Boil bark and wait for solution to cool; add soda ash, feed animal 700 mL of solution 1×day for 2 days without water

TBD=Tick-borne diseases, LSD=Lumpy skin disease, ECF=East Coast Fever

### Mode of remedy preparation, form of use, dosage, and effectiveness

**Figure-1:**
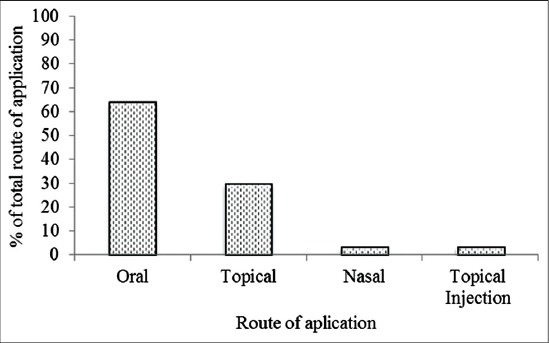
Route of administration for traditional tick-borne disease treatments by Maasai in Northern Tanzania.

Fifty-nine different treatments for TBDs were mentioned. There were 8 treatments for Anaplasmosis, 5 for Brucellosis, 6 for corridor disease, 9 for ECF, 5 for heartwater, 14 for LSD, 8 for red water, and 4 for sweating sickness (Tables-3 and 4). The majority of treatments were prepared in the form of a solution (64%), and topical pastes (12%), topical ointments (10%), pounds (10%), and smoke inhalation (5%). Most medicines were administered orally (68%), topically 24% or through injection into the skin or nasal cavity (8%) (Figure-1). In most cases, the treatments were said to be mostly (59.4%) moderately effective, compared to 17.9% effective and 22.7% very effective (*χ*^2^=30, *df*=2, p<0.001). The dominant animal products used were sheep fat, cattle dung, and cattle ghee. Termite soil, engine oil, salt, and sugar were also used (Table-3). A hot iron was commonly used to burn lesions from LSDs.

**Table-4:** Non-plant materials used to treatment TBDs by Maasai people.

Disease	Material used	Parts used, preparation, dosage, and mentioned efficacy
Corridor disease	Sheep fat	Boil sheep fat, apply the ointment topically around mouth, moderately effective
LSD	Salt and sugar	Wash wounds with water, put salt and sugar paste on wound burn with hot iron, very effective
	Sheep fat	Make a circle around lesion with hot iron, put sheep fat ointment on lesion, burn lesion, once per spot, moderately effective
	Sugar	Put sugar paste on affected skin and burn wound, moderately effective
	Termite soil and ghee	Wash wound, put termite soil and water paste onto wound, smear on ghee, burn with iron, treat until healed, very effective
	Fresh cattle dung	Spread cattle feces over spot, perform once per spot, moderately effective
	Sheep fat	Boil sheep fat to liquefy, inject into spot, perform once per spot, moderately effective
	Car engine oil	Rub oil on cows back, once a day until healed, moderately effective
	Ghee	Wash wound with water, mix soil with water, press on wound, smear ghee on top, burn with hot iron, moderately effective

TBD=Tick borne diseases, LSD=Lumpy skin disease

## Discussion

### Traditional knowledge on tick ecology among the Maasai

Of the 60 different tick species found in Tanzania, only 4 of them are known vectors for TBDs among Maasai livestock [4,20]. In this study, 6 different tick species were identified and had three *maa* names. The tick naming is based on the tick’s coloration. The brown, red, and red-legged ticks are referred to as *Armaheriodo*, the bont, and bont-legged ticks are both striped and are called *Armaheripus*. Similarly, the blue tick is referred to as *Endenuri* or *Armaherikima* because of its bluish gray coloration. The blue tick (*Boophilus decoloratus*) and the brown/red tick (*Rhipicephalus appendiculatus* and *Rhipicephalus* spp.), however, are sometimes confused [21]. The red-legged tick, *Rhipicephalus evertsi evertsi*, was said not to transmit any of the diseases although research suggests its is a vector for ECF [22]. Ticks were said to be more abundant in the dry season. Tick activity increases during the dry season and lulls in the wet months [22]. In addition, increase in TBD instances in the dry season could be due to compromised host immunity as food resources are limited and livestock condition is poor during this time [23]. Furthermore, during the dry season, when water and food are scarce, livestock usually travel further [24], and hence may be exposed more to ticks. Shade is essential in the heat of the dry season, which puts livestock at risk of contracting bont or bont-legged ticks that dwell in the shade or brown ticks that prefer to hide on the bark of trees [22,24]. Feeding site attachment of ticks on livestock could be related to mouth morphology. Both bont tick species have long mouthparts and prefer hairless, tougher skin areas, whereas the blue tick and brown ticks have short to medium mouthparts and attach to softer body parts [22]. However, niche segregation of East African ticks is not fully understood [25].

### Knowledge on TBD and tick control

The traditional tick control methods included burning of old/long grass to kill ticks, applying fresh manure to attachment sites in order to suffocate ticks, hand picking of the ticks and burning them. Kerosene/oil was said to be applied on tick infected areas and the ticks would fall off. The traditional tick control methods the Maasai used are similar to those used by other ethnicities, e.g., the Bukusu of Western Kenya handpick ticks and burn them off, smoke cattle sheds to make ticks fall off, burn tick-infested pastures, and use ethnobotanical products to control ticks [26]. Indeed, hand picking of ticks, application of oil, and pasture burning are common tick control methods across Africa [27]. Considering that 50% of the respondents correctly related specific ticks with the specific disease they transmit indicates that knowledge of tick ecology is relatively good. In other studies, up to 57% of Maasai livestock keepers in Kenya associated ticks with ECF [5]. Consequently, it is likely that due to the Maasai people’s reliance on livestock and their strong cultural attachment, they still have a fair knowledge of TBDs. However, only 7.5%, of interviewed Maasai associated ticks with ECF [28]. The failure to link some of the diseases with tick vectors, e.g., red water [29] shows that traditional knowledge may be limited for disease diagnosis.

### Important medicinal plants for treatment of TBDs

A total of 25 different plant species were used as ethnoveterinary medicine among Maasai. This is similar to other parts of Africa. For example, some farmers in South Africa use herbal remedies over conventional medicine to treat TBDs [30]. In the Kochore district of Gedeo Zone in Ethiopia, 40 plant species are used for ethnoveterinary purposes; 2 of these plants were used to treat TBDs [31]. In the Acholi Subregion of Uganda, 13 plant species belonging to 8 different families were said to control ticks in the area [32].

The *Fabaceae* family is widely used for ethnoveterinary medicine in Africa, e.g., among the Meru in Kenya, [33], Nhema in Zimbabwe, [34] and Venda of South Africa [35]. Overall, the development of traditional disease remedies has developed around plants that are readily and consistently available [33]. In our study, *Aloe* species were the most commonly mentioned plants utilized by the Maasai and were used for the treatment of six of the eight TBDs. Components of *Aloe* plants soothe external wounds and lesions such as those caused by LSD and can reduce internal inflammations [36]. LSD had the most treatment options mentioned, a result consistent with previous research that supports skin irritating ailments as being readily treatable [36].

The three most important plants for treatment of TBDs were *Aloe volkensii*, *Cissus grandifolia*, and *Terminalia brownii*. Plants of the genus *Aloe* have been used traditionally worldwide as a medicinal treatment for a wide variety of ailments due to their biologically active ingredients [37]. The compound aloin and its metabolic derivatives are effective against *Trypanosoma congolese* [38]. Hence, it may be worthwhile assessing its effectiveness against other protozoa-caused diseases, such as ECF and red water.

Plants of the *Vitaceae* family have also been investigated for anti-protozoal properties. *Cissus multistriata* was found to be effective against *Trypanosoma brucei brucei* [39]. The *Combretaceae* family has potentially active compounds such as polyphenols, flavonoids, tannins, saponins, and phytosterols, [39]. *Terminalia brownii* has been found to have antioxidant activities and to act as a hepatoprotective agent [40]. This is important as many TBDs can cause multi-system organ failure including liver damage [41]. Other *Terminalia* spp. and members of the *Combretaceae* family have been found to have anti-bacterial [42] and anti-trypanosomal compounds [43], suggesting that these plants could be of importance in modern pharmacology.

### Plant growth form, plant parts used, and plant availability

The bark from different trees was used to treat seven of the eight TBDs. Trees (60%) and shrubs (22%) were the common growth forms used for making traditional remedies. However, other studies show that herbs are the more common medicinal plant form used in herbal remedies [44,45]. This could be because of the strong seasonality of rainfall in Northern Tanzania, which hinders the growth of many species of herbaceous plants during the dry season. Woody plant forms are easily accessible and readily available for use year round [46]. Plant leaves (30%) were the most common part used. Leaves of 5 different plants were said to treat sweating sickness, heart water, ECF, LSD, corridor disease, anaplasmosis, and red water. Leaves of trees and succulent plants are readily available and easy to access. Use of leaves as the dominant part used is consistent with other research conducted throughout East Africa. Leaves often contain active chemicals and leaf harvesting does not inhibit the growth or survival of the plant species [45]. However, plant and plant part availability varies seasonally [33,47]

### Mode of remedy preparation, form of use, dosage, and effectiveness

Most of the treatments were solutions prepared by boiling plant parts and then administering the solution to the animal orally. Other oral treatments commonly included mashing parts of plants, and administering the decoction to the animals. In general, the type of disease determines which application mode livestock keepers used for remedies. Topical applications were used for skin conditions and mashes and decoctions for internal ailments. The most common method of preparation was often boiling or soaking the plant in water. This allows time for the active ingredients to infuse into the water, thus detaching the chemicals and making the solution potent [48]. Furthermore, liquid solutions were easy to prepare without special equipment or advanced technology [36]. Mostly, 1-2 liters of oral solutions were reported to be administered to livestock; the frequency and duration of the treatments varied. While these are relatively large volumes, traditional treatments rarely have side effects [49]. Most traditional remedies are limited to trial and error [48] and thus variation in dosages can be due to season harvested, as chemical concentrations in plants vary by season [50]. Some of the plants have a strong bitter taste and are only administered in small dosages. Others are considered too “strong” and only given to livestock in small amounts. Livestock keepers also adjust dosages according to the size of the animal [33,35]. Other factors may be age and body condition, perceived “severity” of illness and the specific remedy availability.

The reported effectiveness varied; however, the remedies were seen to be moderate to highly effective in treating TBDs. Most treatments for heartwater were seen to be ineffective while treatments for red water and anaplasmosis were often said to be effective. The efficacy of treatments for ECF and sweating sickness were said to depend on the stage of the disease. Often, traditional treatments are viewed as just as effective as modern medicine [51] and indeed laboratory experiments have confirmed the efficacy of certain plant families against bacteria and protozoans *in vitro* [42].

## Conclusion and Relevance

The Maasai people interviewed could identify 6 tick species and knew about 8 TBDs. There were 25 species of plants identified and used in 59 treatments of TBDs among livestock. Many of the plant species and families used for TBD treatment (*Aloe volkensii, Cissus grandifolia, Terminalia brownie*; Families: *Fabaceae*, *Euphorbiaceae*, *Solanaceae*) are known to have pharmacological importance, further validating their credibility as ethnoveterinary medicine. This study emphasizes a need for an integrated approach in livestock health care in managing TBDs. The high use of traditional ethnoveterinary remedies highlights the need to support this as livestock health care practice. Rural based ethno-veterinary centers should be established and be able to investigate and confirm plants and treatments and standardize dosages, in order to maximize effectiveness.

## Authors’ Contributions

JK designed the study and helped in data collection, data analysis and manuscript preparation. JB and AS helped in data collection and in the initial manuscript preparation. CK provided useful scientific and technical input on the manuscript. All authors read and approved the final manuscript.
